# Changes of microbiota level in urinary tract infections: A meta-analysis

**DOI:** 10.1515/med-2023-0702

**Published:** 2023-05-26

**Authors:** Xia Weng, Yajun Liu, Haiping Hu, Meichai Wang, Xiaoqin Huang

**Affiliations:** Urology Department, Zhejiang Hospital, Hangzhou 310013, Zhejiang Province, China; Orthopedics Department, Zhejiang Hospital, No. 1229, Gudun Road, Hangzhou 310013, Zhejiang Province, China; Neurosurgery Department, Zhejiang Provincial Hospital of Traditional Chinese Medicine, Hangzhou, Zhejiang Province, China

**Keywords:** Urinary tract infections, microbiota, *Escherichia coli*, *Lactobacillus*, *Enterobacteriaceae*

## Abstract

No consensus has been reached on the dysbiosis signs of microbiota in patients with urinary tract infections (UTIs). This meta-analysis aimed to verify the relationship between microbiota levels and UTIs. PubMed, Web of Science, and Embase databases were retrieved for related articles published from inception until October 20, 2021. The standardized mean difference (SMD) and its related 95% confidence intervals (CIs) of the microbiota diversity and abundance were pooled under a random-effects model. Twelve studies were included in this meta-analysis. The pooled analysis revealed that the microbiota diversity was lower in patients with UTIs than in healthy individuals (SMD = −0.655, 95% CI = −1.290, −0.021, *I* ^2^ = 81.0%, *P* = 0.043). The abundance of specific bacteria was higher in UTI subjects compared with healthy control individuals (SMD = 0.41, 95% CI = 0.07–0.74, *P* = 0.017), especially in North America patients with UTIs. Similar results were also found in studies with the total sample size being greater than 30. Importantly, *Escherichia coli* levels were increased in patients with UTI, whereas *Lactobacillus* levels decreased. *E. coli* and *Lactobacilli* have huge prospects as potential microbiota markers in the treatment of UTIs.

## 1 Introduction

Urinary tract infections (UTIs) are among the most common infectious diseases worldwide [1] and are often community- or hospital-acquired [2,3]. According to previous reports, nearly 50% of the population will experience UTIs throughout their lives, costing US $3.5 billion in health care annually [4,5,6]. UTIs are prevalent in older women, particularly in 8–10% of postmenopausal women [7]. Moreover, almost half of the women with their first episode of a UTI experience a recurrence, which occurs in 5% of UTI cases [8]. Furthermore, it has been reported that up to 8% of children will suffer from at least one UTI between 1 month and 11 years [9]. Therefore, providing accurate prediction, timely diagnosis and treatment, and exploring the mechanism of UTIs is essential.

The microbiota is widely considered an essential “second genome” that matures throughout childhood development and contributes to body health [1,10,11]. It has long been thought that urine is sterile in healthy individuals; however, the Human Microbiome Project later demonstrated the presence of a bladder and urinary microbiome [12]. Multiple infections, such as UTIs, lung disorders, and influenza, are associated with microbiome dysbiosis [13,14]. Complex microbial communities are also important for diagnosing different diseases and planning individual drug treatments for patients [12]. Next-generation sequencing had been widely used for diagnostics in UTIs. Current evidence suggests that the 16S ribosomal RNA (rRNA) gene is highly conserved and unique amongst bacteria, given its essential functions [15]. The combination of PCR detection and sequence analysis has gained significant momentum in recent years for identifying unknown bacterial species [16]. In contrast, metagenomic sequencing can analyze broader populations of microbial communities in clinical samples and observe microbiota dynamics under different clinical conditions [11]. Several studies have illustrated the associations between changes in the urine microbiome using high-throughput sequencing in patients with lower urinary tract symptoms, urge incontinence, and bladder cancer [17,18,19]. Over the years, *Lactobacillus*, *Gardnerella*, *Streptococcus*, *Staphylococcus*, and *Corynebacterium* have been confirmed as pathogenic bacteria in UTIs [15]. Furthermore, the urine microbiome diversity has been documented in female patients with acute uncomplicated cystitis and recurrent cystitis [12]. In addition, Horwitz et al. revealed that patients with decreased microbiome diversity were more frequently to have UTIs after the insertion of an indwelling catheter [20]. Conversely, another report described no significant changes in microbiome diversity between patients with asymptomatic pyuria and neurogenic bladder [21]. Meanwhile, it had been reported that there was no significant difference in microbiome diversity between patients with recurrent urinary tract infections (rUTIs) and healthy individuals [7]. Specifically, the abundance of *Lactobacillus* species was found to be similar in both subjects [7]. However, a different report showed that *Lactobacillus* levels were significantly decreased in patients with rUTIs [22].

Accordingly, we conducted a meta-analysis to systematically describe the dysbiosis signs of the microbiota in patients with UTIs. Indeed, a meta-analysis can reduce the heterogeneity among studies by pooling a large amount of available data and providing more precise estimates.

## 2 Materials and methods

### 2.1 Search strategy

The present meta-analysis was designed following the Preferred Reporting Items for Systematic Reviews and Meta-analysis (PRISMA) 2020 statement. Two authors independently conducted a systematic literature search in online databases, including PubMed, ISI Web of Science, Embase, Google Scholar, PMC, and CNKI (Chinese National Knowledge Infrastructure) from inception until Oct 20, 2021. The literature language was limited to English and Chinese. The literature was searched in each database by using a particular search strategy. For instance, MeSH terms and the following keywords were used in PubMed: (microbiota[Mesh Terms] OR microbiome [Mesh Terms] OR “microbiota*” [textword] OR “microbiome*” [text word] OR “bacteria” [Mesh Terms] OR “bacteria*” [textword] AND (“urinary tract infection”[MeSH Terms] OR “urinary tract infection*”[Text Word] OR “Bacteriuria” [Mesh Terms] OR “Bacteriuria” [Text Word]). Case reports, comments, letters, and review articles were excluded during the search process. Relevant studies cited in the included or excluded articles were also manually searched to assess their eligibility.

### 2.2 Inclusion and exclusion criteria

Two authors independently reviewed the titles and abstracts of the searched articles, and then the full text was reviewed to decide whether they met the inclusion criteria. Case–control studies and cohort studies or other types of clinical studies about microbiota diversity or abundance association with UTI were included. The inclusion criteria were as follows: (1) subjects with UTI-related symptoms; (2) the diagnosis of UTI was clinically confirmed according to current guidelines; (3) healthy individuals were included as a control group; (4) microbiota diversity or abundance related to UTI was detected in subjects with UTI diseases and the control group; (5) metagenomics, metatranscriptomics, host transcriptome, or 16S gene RNA sequencing was used to detect microbiota diversity and abundance; (6) the sample size of the case and control groups was provided; (7) studies were published in Chinese or English. Studies were excluded if they were (1) case reports, comments, animal or cell line studies, or review articles; (2) duplicate articles; (3) articles with no data on the microbiota diversity or abundance in patients and control subjects; (4) articles not related to metagenomics, metatranscriptomics, host transcriptome, or 16S gene RNA sequence; and (5) articles not related to the microbiome.

### 2.3 Data extraction and quality assessment

All screened articles that met the inclusion criteria underwent data extraction by two authors. The following details were extracted from the included manuscript: first author, year of publication, distinct in where study conducted, study design, ages of subjects, the number of patients and controls, disease duration, sample collection method, sample type, comorbidities, microbiota detection methods, mean and SD or SE value of microbiota diversity or abundance of both patients and controls. Any disagreements were resolved by the third author until a consensus was reached.

In addition, the quality assessment of studies was conducted by the Newcastle–Ottawa Scale (NOS) tool [23], which could be used to assess the risk of bias in all included case–control studies or cohort studies. The NOS comprises eight items, categorized into three dimensions: selection, comparability, and depending on the study type-outcome (cohort studies) or exposure (case–control studies). The total quality score ranged from 0 to 9; a high-quality study was associated with a higher score. Studies with scores lower than 5 will be excluded.

### 2.4 Statistical analysis

The mean and SD values of the diversity or abundance of microbiota in patients and control subjects were extracted. The combined effect was represented as standardized mean difference (SMD) and its 95% confidence intervals (CIs). Heterogeneity was statistically calculated by the Cochrane *Q* test (*P* < 0.10) and the *I* ^2^ statistic [24] to estimate the heterogeneity across studies. *I* ^2^ values of 0 to 25%, 25 to 50%, 50 to 75%, and 75% indicated insignificant, low, moderate, and high heterogeneity, respectively. The pooled effect was combined under the random-effects model when the *I* ^2^ value was >75%. Otherwise, a fixed-effects model was used to calculate the SMD and its 95% CIs. To address the potential sources of heterogeneity, subgroup analysis was also performed based on the difference in age, sample size (the sum of case group and control group) >30 or not, sample type, and microbiome type. The sensitivity analysis and publication bias were performed to evaluate the influence of each study. Begg’s or Egger’s tests were used to evaluate the publication bias. Stata software version 12.0 (Stata Corp. LP, TX, USA) was used to perform the above statistical analyses. A *P* value of <0.05 was statistically significant.

## 3 Results

### 3.1 Clinical characteristics and quality of the included studies and subjects

**Figure 1:**
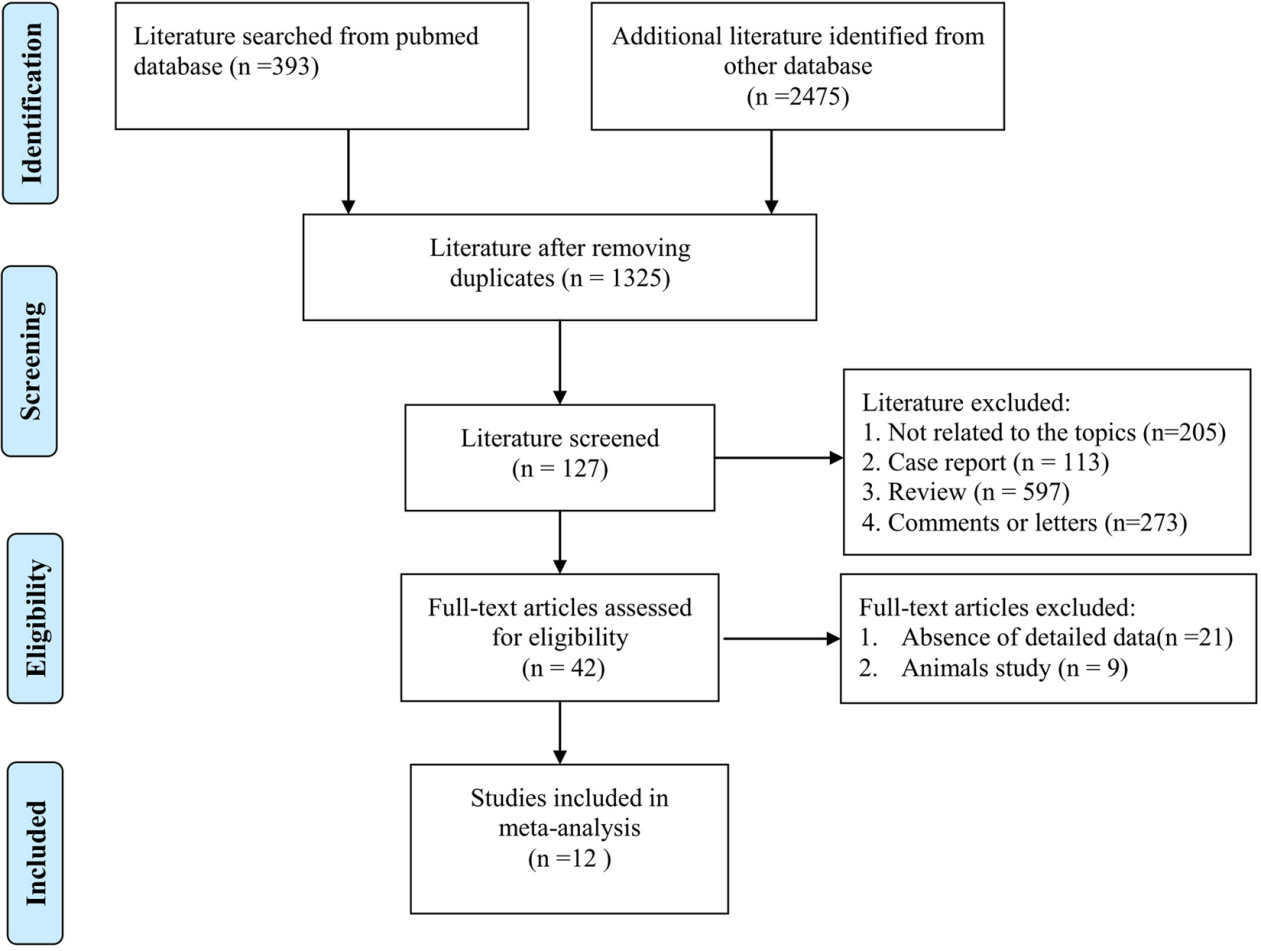
The diagram flowchart for selecting the included studies.

Our literature search yielded a total of 2,868 articles from PubMed, Web of Science, Embase Google Scholar, PMC, and CNKI (Chinese National Knowledge Infrastructure). After removing duplicates, 1,325 articles were screened inclusion. After removing the literature that was not related to the topics, there were 127 articles to be carefully read. Then, 85 articles were further removed. Then, 30 articles were deleted for lack of detailed data to calculate the pooled mean and SD values. Ultimately, 12 articles met the inclusion criteria. The detailed literature searching diagram is shown in Figure 1. The results of the NOS quality score of the included studies ranged from 6 to 9 (Table 1).

**Table 1:** Clinical characteristics of the included articles

First author	Year	Country	Study design	Study group	No.	Age (years)	Sample collection	Sample type	Complicated disease	Microbiota type	Detection method	NOS score
Breffini	2021	Canada	Cohort study	rUTIs	17	65.47(9.05)^a^	Catheter	Urine	Diabetes	*F. magna*	PCR, 16S rRNA gene	7
				HC	20	65.2(7.35)^a^			Renal calculi	*Klebsiella aerogenes*		
Zhu	2021	China	Case–control study	rUTIs	16	61.56(7.08)^a^	Catheter	Urine	N/A	*Bacteroidetes*	PCR, 16S rRNA gene	7
				HC	8	58.13(6.15)^a^						
Monique	2021	USA	Case–control study	rUTIs	24	71.2(8.7)^a^	Catheter	Urine	Diabetes	*Lactobacillaceae*	PCR, 16S rRNA gene	8
										*Aerococcace*		
				HC	23	69.3(6.6)^a^				*Enterobcteriaceae*		
Andrea	2020	USA	Case–control study	UTIs	42	About 60	Catheter	Urine	N/A	*Escherichia coli*	PCR, 16S rRNA gene	6
				HC	6	About 60						
Catherine	2020	USA	Cross-sectional study	UTIs	11	11.0(6)^a^	Catheter	Urine	Neuropathic bladder	*Enterobacteriaceae*	PCR, 16S rRNA gene	6
				HC	4	15.0(6)^a^				*Staphylococcus*		
Lauren	2020	USA	Cross-sectional study	UTIs	9	<48 M	Catheter	Urine	N/A	*Escherichia coli*	PCR, 16S rRNA gene	7
				HC	76	<48 M						
Krystal	2018	USA	Cohort study	UTIs	69	62(37–85)^b^	Catheter	Urine	Hypertension	*Lactobacillus*	PCR, 16S rRNA gene	7
										*Peptonlphllus*		
										*Bacteroides coagulans*		

										*Bacteroides fragilis*		
										*β-Proteobacteria*		
				HC	30	57(38−50)^b^			Coronary artery disease			
Niko	2018	Finland	Case–control study	UTIs	37	20.3(27.2)^a^ M	Catheter	Stool	N/A	*Antimicrobials*	PCR, 16S rRNA gene	9
				HC	69	21.8(30.6)^a^ M						
Casper	2016	Netherlands	Cohort study	UTIs	10	63.4–64.3^c^	Catheter	Stool	N/A	*Fusobacteria*	PCR, 16S rRNA gene	8
				HC	10					*Bacteroidetes*		
Deborah	2015	USA	Cohort study	UTIs	18	70.9(57–88)^b^	Catheter	Urine	N/A	*Escherichia coli*	PCR, 16S rRNA gene	6
				HC	8	N/A						
Tasha	2015	USA	Case–control study	UTIs	10	50.80(20.11)^a^	Catheter	Urine	N/A	*Proteobacteria*	PCR, 16S rRNA gene	6
										*Bacteroidetes*		
										*Firmicutes*		
				HC	10	50.90(21.9)^a^				*Verrucomicrobia*		
Jascha	2015	Poland	Cohort study	UTIs	8	About 60	Stool sample	Stool	N/A	*Firmicutes*	PCR, 16S rRNA gene	6
										*Verrucomicrobia*		
										*Proteobacteria*		
				HC	5	About 60				*Actinobacteria*		

Note: a: mean (SD); b: median (range); c: range; M: month; rUTIs: recurrent urinary tract infections; HC: health control; N/A: not unavailable; NOS: Newcastle–Ottawa Scale; PCR: polymerase chain reaction.

**Figure 2:**
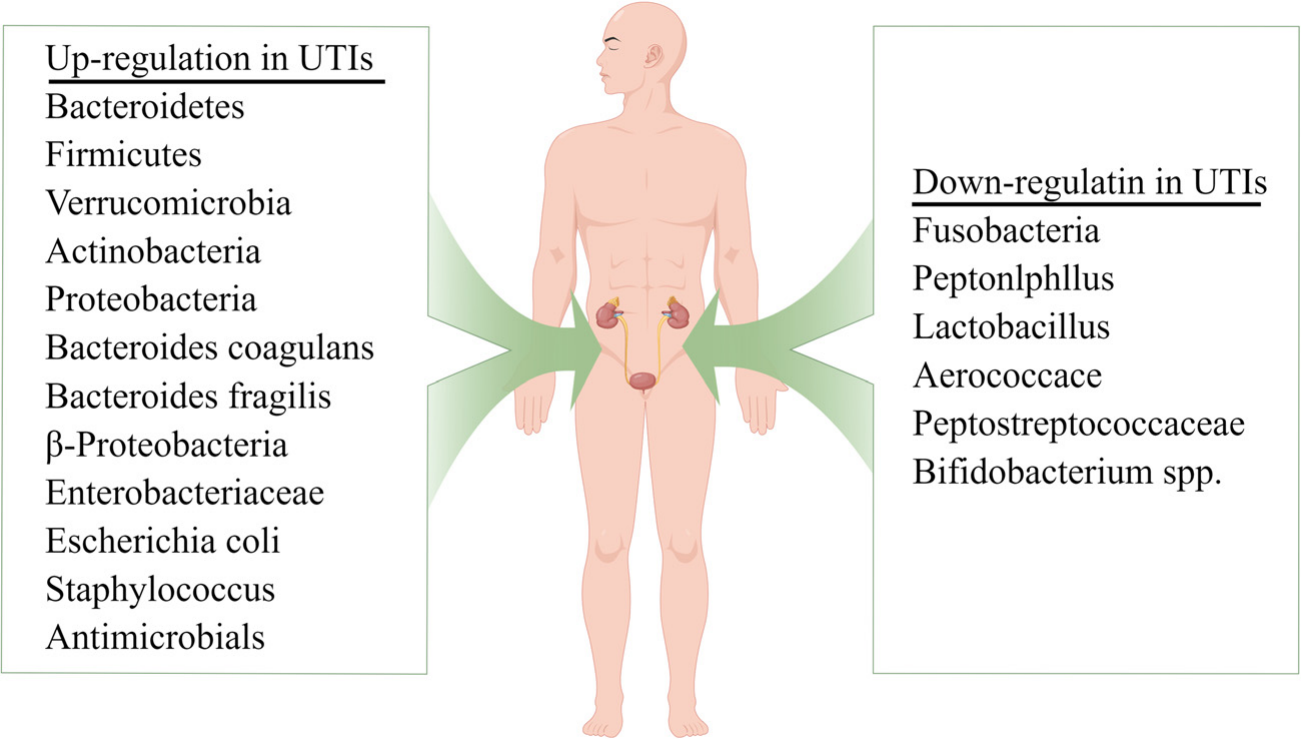
Abnormal alteration of microbiota in UTIs or rUTIs patients of the included studies.

The clinical characteristics of patients in the included studies are presented in Table 1. The included studies involved a total of 1,888 individuals (956 patients and 932 healthy control individuals). These studies were published from 2015 to 2021 and conducted in North America (Canada and the USA) [7,20,25,26,27,28,29,30], Europe (Finland, Poland, and the Netherlands) [8,31,32], and Asia (China) [33]. The study designs included case–control (*n* = 5), cohort study (*n* = 5), and cross-sectional (*n* = 2) studies. The study subjects were children (age range: 20.3 months to 11 years) [27,29,32] and older adults (age range: 50–72 years) [7,8,20,25,26,28,30,31,33]. Some of the patients with UTI suffered comorbidities, including diabetes, renal calculi [7], neuropathic bladder [27], hypertension, coronary artery disease [26], and so on. Moreover, the sample size of these included studies (case group and control group) varied from 13 to 99. The sample types were urine or stool; the urine was collected by a urethral catheter. 16S rRNA gene sequence technology was used to detect the microbiome diversity and abundance. DNA was extracted from the microbiota, and the abundance of each microbiota was detected by quantitative reverse transcription polymerase chain reaction (qRT-PCR). Moreover, given that the included studies may provide different microbiota diversity or abundance data, we treated each microbiota as an independent group during the meta-analysis. Figure 2 illustrates the upregulated and downregulated microbiota in the included studies. Four studies illustrated the association between *Bacteroidetes* and UTIs [8,25,32,33], and four studies presented the relationship between *Proteobacteria* and UTI [25,26,31,32]. In addition, as shown in Figure 2, *Lactobacillus* was downregulated in patients with UTIs. *Escherichia coli*, *Firmicutes*, *Verrucomicrobia*, and *Enterobacteriaceae* were upregulated in patients with UTIs.

### 3.2 Random effects meta-analysis: the microbiota diversity in patients with UTIs

**Figure 3:**
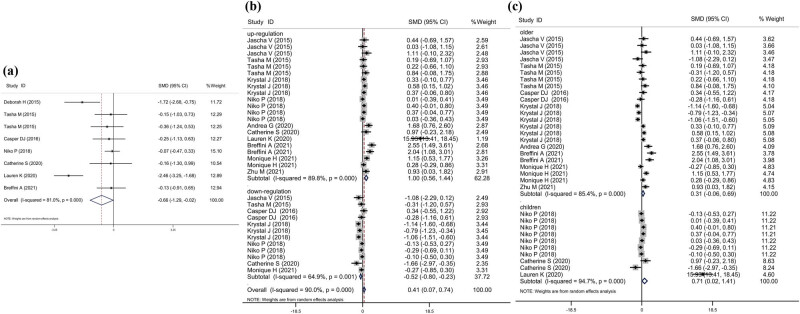
Forest plots of the relationship between microbiota expression level and patients with UTI. (a) The forest plots of the alteration in alpha diversity of microbiome in patients with UTI. (b) This is the overall analysis and subgroup analysis based on the increasing or not of the abundance of microbiota in patients. For each study, the estimate of mean abundance of each microbiota difference and its 95% CI is plotted with a diamond. SMD, standard mean difference; Chi^2^, chi square statistic; df, degrees of freedom; *I* ^2^, *I*-square heterogeneity statistic. (c) Subgroup analysis of the association between microbiota abundance and patients according to the difference of patient age.

Eight studies [7,8,20,25,28,29,32,34] examined the association between microbiota diversity and UTIs, including 154 patients with UTI and 203 healthy individuals. It has been established that there is a significant difference in bacterial richness of the urine microbiota between patients with UTI who developed a symptomatic infection and healthy control individuals [32]. Usually, alpha diversity is measured by the Shannon Diversity or Chao1 Index. Many studies reported the Shannon Diversity Index to assess microbiota diversity in the present meta-analysis. The pooled relative risk for UTI incidence was −0.66 (95% CI −1.29 to −0.02, *I* ^*2*^ = 36.77%, *P* = 0.043), indicating a significant inverse association between UTI and microbiota alpha diversity (Figure 3a).

### 3.3 Random effects meta-analysis: the microbiota abundance in patients with UTI

Twelve studies, including 33 comparisons between 956 patients with UTI and 932 healthy individuals, were used to evaluate the change of microbiota abundance during UTI. Overall, the abundance of microbiota was higher in UTI subjects (SMD = 0.41, 95% CI = 0.07–0.74, *I* ^*2*^ = 90.0%, *P* = 0.017, Figure 3b). Among these 33 comparisons, 21 described the increasing trend of microbiota abundance between patients with UTI and healthy controls (SMD = 1.00, 95% CI = 0.56–1.44, *I* ^*2*^ = 89.8%, *P* < 0.01, Figure 3b). In addition, 12 comparisons reported decreased microbiota abundance between patients with UTI and healthy controls (SMD = −0.52, 95% CI = −0.80, −0.23, *I* ^*2*^ = 64.9%, *P* < 0.01, Figure 3b).

### 3.4 Subgroup meta-analysis: the microbiota abundance in patients with UTI

In the present meta-analysis, Cochran’s *Q* test and *I* ^2^ test were used for heterogeneity analysis across the included studies. Our findings demonstrated that significant heterogeneity existed across these included studies. Therefore, we conducted a subgroup analysis to explore the potential sources of heterogeneity. Factors, including age, distinct in where conducted, sample size, and difference in microbiota abundance, were used to perform subgroup analysis in Stata software.

**Figure 4:**
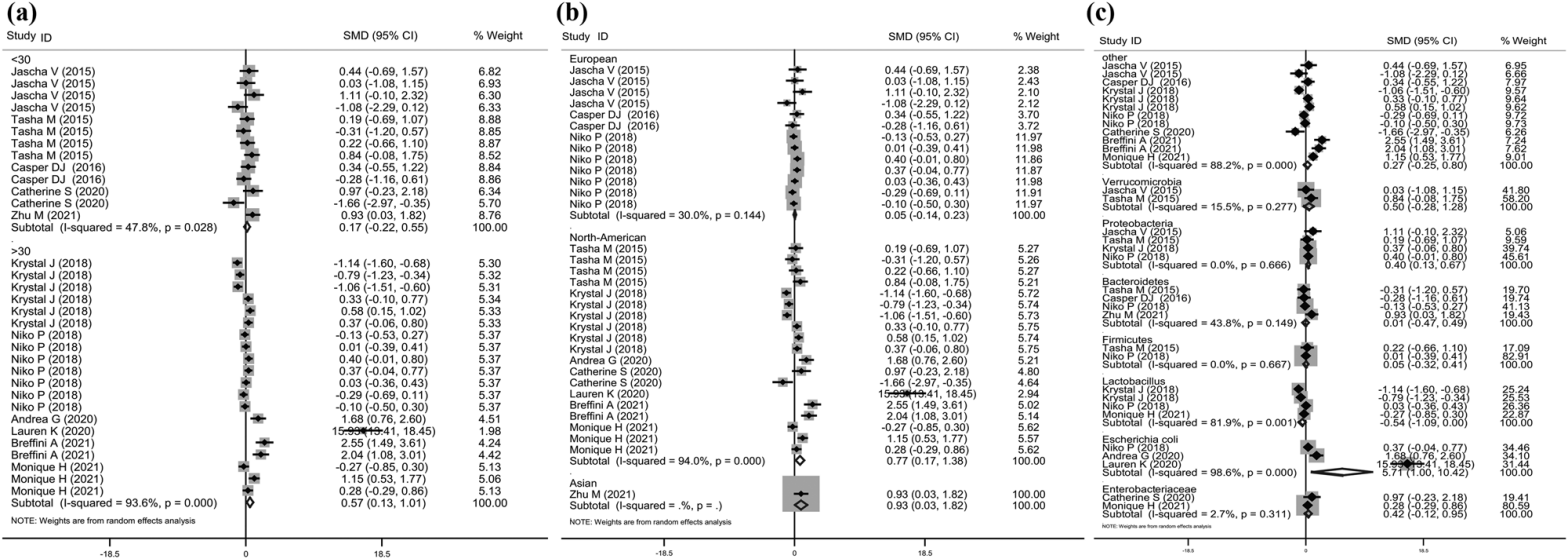
Subgroup analysis of the association between microbiota abundance and patients with UTI based on the different variation. (a) Forest plot analysis according to the difference in distinct of the included studies. (b) Forest plot analysis according to the difference in sample size of the included studies. (c) Forest plot analysis according to the difference in microbiota genera.

After pooling data from 23 comparisons, including older participants, no significant increase in the abundance of microbiota was found in patients with UTI (SMD = 0.31, 95% CI = −0.06, −0.69, *I* ^*2*^ = 85.4%, *P* = 0.099, Figure 3c). However, a significant increase in microbiota abundance in children was observed from ten comparisons (SMD = 0.71, 95% CI = 0.02, −1.41, *I* ^*2*^ = 94.7%, *P* = 0.045, Figure 3c). Moreover, 13, 19, and 1 comparison were performed in Europe, North-America, and Asia, respectively. Results indicated that increased microbiota abundance was significantly associated with patients with UTI in North America (SMD = 0.77, 95% CI = 0.17–1.38, *I* ^*2*^ = 94.0%, *P* = 0.013, Figure 4a). However, there was no significant association between microbiota abundance and UTI conducted in Europe (SMD = 0.05, 95% CI = −0.14−0.23, *I* ^*2*^ = 30.0%, *P* = 0.610, Figure 4a) and Asia. Moreover, there were 13 comparisons with a sample size of <30 to illustrate the change in microbiota abundance in patients with UTI. However, there was no significant increase or decrease in microbiota abundance (SMD = 0.17, 95% CI = −0.22–0.55, *I* ^*2*^ = 47.8%, *P* = 0.396, Figure 4b). Pooling of 20 comparisons that involved a sample size >30 showed a significant increase in microbiota abundance (SMD = 0.57, 95% CI = 0.13–1.01, *I* ^*2*^ = 93.6%, *P* = 0.011, Figure 4b).

Finally, the change in microbiota abundance between patients with UTI and healthy participants was investigated. As shown in Figure 4c, *Proteobacteria* (SMD = 0.40, 95% CI = 0.13–0.67, *P* = 0.004) showed a significant increasing trend in patients with UTI compared with healthy controls when the data from four studies [25,26,31,32] were pooled. Similarly, *E. coli* (SMD = 5.71, 95% CI = 1.00–1.42, *I* ^2^ = 98.6%, *P* = 0.018) was also increased in patients with UTI [28,29,32]. However, *Lactobacillus* (SMD = −0.54, 95% CI = −1.09 to 0.00, *I* ^*2*^ = 81.9%, *P* = 0.051) exhibited a decreasing trend in patients with UTI compared with healthy controls in three studies [26,30,32]. No significant evidence was found on the association between UTIs and the abundance change of *Bacteroidetes*, *Enterobacteriaceae*, *Firmicutes*, and *Verrucomicrobia*, but with an increasing trend in patients with UTIs.

### 3.5 Sensitivity analyses and the evaluation of publication bias

**Figure 5:**
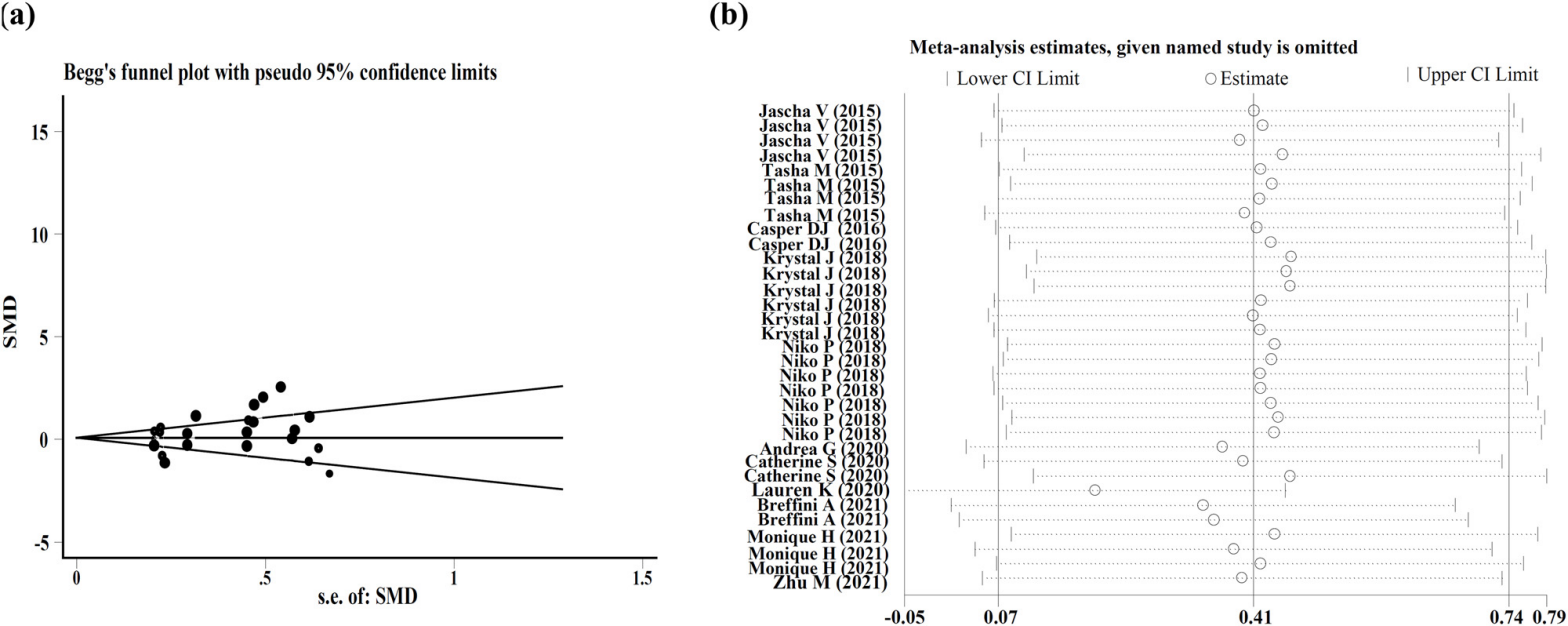
The publication bias and sensitivity analysis of the included studies. (a) Funnel plot of publication bias from Begg’s test. SE, standard error; SMD, standardized mean difference. (b) Sensitivity analysis. There were no studies that fell outside of the lower or upper limit.

As shown in Figure 5b, no comparisons were out of the lower and upper limits after sensitivity analysis. The funnel plot and Egger’s and Begg’s tests were used to assess publication bias. As shown in Figure 5a, the funnel plot exhibited slight asymmetry. Although a statistically significant *P* value was obtained from Begg’s test (0.670), the *P* value of Egger’s test was significant (0.010). Besides the small sample size, the funnel plot asymmetry could be due to other factors, including heterogeneity, data irregularities, and selection bias [35].

## 4 Discussion

It is widely acknowledged that UTIs are caused by ascending infections by bacteria outside the urinary tract or from reinfection by intracellular bacterial communities within the urothelium [30]. The urinary microbiota and uropathogens are widely thought to have a commensal relationship in UTIs [30]. Therefore, over the years, significant emphasis has been placed on the urinary microbiome’s role in UTIs and rUTIs [1,7,10,30,36]. This meta-analysis sought to assess recent studies which evaluated different microbiota in the urogenital system. Importantly, our results contribute to a better understanding of the change in microbiota abundance and the role of microbiota in patients with UTIs or rUTIs. Here, we retrieved all available urinary microbiome studies and integrated the data from 12 articles involving 1,888 individuals (956 patients and 932 healthy control individuals). To the best of our knowledge, this is the first study to indicate that patients with UTIs have lower microbial diversity than healthy individuals. The abundance of *Proteobacteria* and *E. coli* was increased in patients with UTI symptoms. However, *Lactobacilli* exhibited a decreasing trend in patients with UTI compared with healthy controls. These findings provided novel insights into the potential of microbial-targeting strategies for treating UTIs.

Many microbiota species have been documented to confer a protective role within healthy hosts [10]. Consistent with the literature, microbial diversity decreased, and microbiome composition changed during a UTI, which has been demonstrated in other systems [37–40]. However, it should be borne in mind that aging by itself results in decreased stability and diversity of the microbiota [41]. Our meta-analysis confirmed this point, and the combined microbiota abundance was higher in children than in older adults. Moreover, it has been reported that peripheral fat could transform into estrogen in obese women [42]. Consequently, the microbiota diversity decreased with increasing BMI resulting from the effect of estrogen on the vagina. Anglim et al. substantiated a significantly lower diversity and richness in microbiota species in obese women patients with rUTIs than in healthy individuals [7]. Furthermore, it has been established that lower UTI was more common in women than in men; several studies have described the change in urine microbiome in urological disorders in women [8,22,28,32,43,44]. Probably, it is highly likely that differences in the urinary microbiota are normally present between males and females. In this respect, Santiago-Rodriguez et al. identified differences in women and men regardless of the infection status [43]. Some of the identified different microbiota may reflect the uniqueness of the female genitourinary tract [45,46]. Fortunately, if the changes in the diversity and abundance of the urinary microbiome could be confirmed prior to UTI occurrence and other diseases, it could be applied to identify high-risk or low-risk microbiome biomarkers. Consequently, future therapeutic plans that target these identification biomarkers could be designed to prevent clinical UTIs.

**Figure 6:**
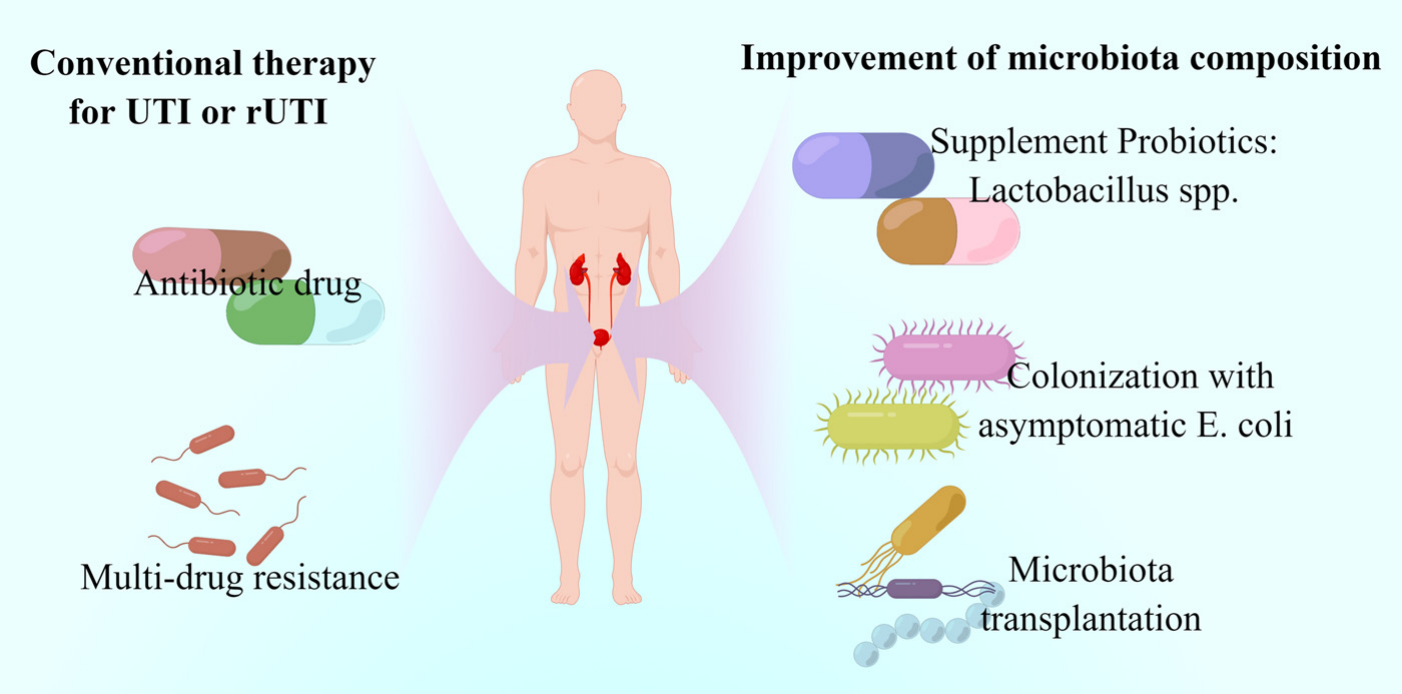
Novel intervention strategy based on modulating the microbiota to treat UTIs.

Herein, we found that the pooled *Lactobacillus* level exhibited a decreasing trend in patients with UTIs. It is well established that *Lactobacilli* play a protective role against uropathogens [12]. Nonetheless, this protective role was limited only to women [46]. Hence, the absence of *Lactobacilli* in the vagina is a risk factor for UTIs, while the probiotic *Lactobacilli* reduce susceptibility to rUTIs [12]. rUTIs samples exhibited a decreased microbial diversity and unchanged abundance of *Lactobacillus* after local estrogen therapy (LET) therapy. Furthermore, ample evidence suggests that *Lactobacilli* can inhibit the growth of *E. coli* [28,47], prevent the colonization of *E. coli* [44], and consequently prevent the occurrence of UTIs. Jung et al. found that women patients with rUTIs without *Lactobacilli* had a fourfold increased risk of *E. coli* colonization compared with healthy women [44]. The specific uropathogenic *E. coli* strain that can induce UTIs is commonly present in feces [48]. Moreover, *E. coli* was once recognized as the most common etiological agent of UTIs, responsible for more than 80% of women patients with UTIs [43]. However, Garretto et al. demonstrated that pathogenic *E. coli* had a weak predictive value for UTIs [28]. Instead, UTIs may result from the interaction of multiple microbiota [28,44]. Our results suggest that prophylactic probiotics, which have been used in gastrointestinal disorders such as diarrhea, colitis, and inflammatory bowel disease, may be used to prevent UTIs or rUTIs to avoid the emergence of multi-antibiotic resistant uropathogens [49]. This treatment differed from conventional drug therapy, which generally reduced multiple drug resistance, as shown in Figure 6. However, supplement with probiotic (such as *Lactobacillus* spp.) or microbiota transplant will overcome the lack of conventional drug treatment. For example, a randomized clinical phase II trial showed that administration of *Lactobacillus crispatus* probiotic (Lactin-V) via intravaginal suppository significantly reduced the occurrence of rUTIs [50]. *E. coli* has also been associated with UTIs in murine models and patients [51]. Attenuated *E. coli* isolates from a mutation in the fimbrial adhesins are reportedly involved in establishing asymptomatic long-term colonization of the urinary tract and outcompeting uropathogens [52]. In addition, patients with UTIs may benefit from fecal microbiota transplantation by decreasing the colonization of antibiotic-resistant pathogens and increasing antibiotic susceptibility of the pathogens, which has been confirmed by Hocquart et al. [53].

Besides *Lactobacilli* and *E. coli*, *K. pneumoniae*, *S. saprophyticus*, and *Strep agalactiae* were also the common infectious agents in UTI [54–56]. For instance, Garretto et al. [28] and Thompson et al. [6] had examined the level of *Staphylococcus* and *Klebsiella* during the occurrence of UTI. In fact, *S. saprophyticus* is second only to *E. coli* as the most frequent infectious agents in UTI for women, especially in young sexually active women [54]. However, from young boys to elderly men, *S. saprophyticus* can cause UTI [57]. In healthy women, Rupp et al. found that *S. saprophyticus* colonized the urogenital tract at a rate of 6.9%, particularly colonizing the rectum at a rate of 40% [58]. *S. saprophyticus* mainly adhere to urothelial cells by means of a surface-associated protein and lipoteichoic acid to demonstrate its virulence [59]. In addition, the huge genes also played an important role in maintaining the virulence of the bacteria strains. For example, *wabG* gene contributed to the virulence of *K. pneumonia* by forming the outer core lipopolysaccharide. The *uge* gene encoded uridine diphosphate galacturonate 4-epimerase that ensures smooth lipopolysaccharide and capsule biosynthesis [55]. Furthermore, UTI caused by *K. pneumonia* was mainly reported in Asian district, such as Taiwan and South Korea [55]. Therefore, patients with UTI should be treated individually based on their sex, age, or distinctive characteristics.

However, our results should be interpreted with caution due to the limitations of the present study. First, the included studies had a small sample size. Accordingly, a larger multi-center study needs to be conducted with more patients to achieve more robust conclusions. Second, the included studies mainly were case–control studies; more randomized clinical trials should be performed and then included in further analysis. Third, both males and females were involved in the included studies, but there may be a significant difference in microbiota abundance and types induced by sex hormone, which could lead to inaccuracy in this analysis. Finally, the included studies characterized the microbiota related to UTIs mainly by 16S rRNA gene sequencing analyses, which did not reveal changes in the metabolic activity of the microbiota communities [36]. Furthermore, due to insufficient data, the changes in the microbiota during UTIs were not analyzed.

## 5 Conclusion

This meta-analysis reveals that patients with UTIs have lower microbiome diversity. A significant increase in the abundance of microbiota in children with UTIs was also observed. Simultaneously, *E. coli* was increased in patients with UTIs. *Lactobacilli* exhibited a decreasing trend in patients with UTIs compared with healthy controls. Therefore, *E. coli* and *Lactobacilli* may be potential microbiota markers in the treatment of UTIs, which provided novel insights into the individual therapy strategies for patients with UTIs. However, the significance of these results is limited by the small number of included studies and the sample size of patients. Therefore, larger studies are warranted before more definitive conclusions can be made.
